# Transcriptome sequencing analyses uncover mechanisms of citrus rootstock seedlings under waterlogging stress

**DOI:** 10.3389/fpls.2023.1198930

**Published:** 2023-05-31

**Authors:** Wen He, Liang Luo, Rui Xie, Jiufeng Chai, Hao Wang, Yan Wang, Qing Chen, Zhiwei Wu, Shaofeng Yang, Mengyao Li, Yuanxiu Lin, Yunting Zhang, Ya Luo, Yong Zhang, Haoru Tang, Xiaorong Wang

**Affiliations:** College of Horticulture, Sichuan Agricultural University, Chengdu, Sichuan, China

**Keywords:** flooding, transcriptomic, citrus rootstock, *Citrus junos* Sieb ex Tanaka cv. Pujiang Xiangcheng, differentially expressed genes

## Abstract

Citrus plants are sensitive to waterlogging, which can cause yield reduction. Their production heavily depends on the rootstock being used for grafting of scion cultivars, and the rootstock is the first organ to be affected by waterlogging stress. However, the underlying molecular mechanisms of waterlogging stress tolerance remain elusive. In this study we investigated the stress response of two waterlogging-tolerant citrus varieties (*Citrus junos* Sieb ex Tanaka cv. Pujiang Xiangcheng and Ziyang Xiangcheng), and one waterlogging-sensitive variety (red tangerine) at the morphological, physiological, and genetic levels in leaf and root tissues of partially submerged plants. The results showed that waterlogging stress significantly decreased the SPAD value and root length but did not obviously affect the stem length and new root numbers. The malondialdehyde (MDA) content and the enzyme activities of superoxide dismutase (SOD), guaiacol peroxidase (POD), and catalase (CAT) were enhanced in the roots. The RNA-seq analysis revealed that the differentially expressed genes (DEGs) were mainly linked to ‘cutin, suberine, and wax biosynthesis’, ‘diterpenoid biosynthesis’, and ‘glycerophospholipid metabolism’ in the leaves, whereas were linked to ‘flavonoid biosynthesis’, ‘biosynthesis of secondary metabolites and metabolic pathways’ in the roots. Finally, we developed a working model based on our results to elucidate the molecular basis of waterlogging-responsive in citrus. Therefore, our data obtained in this study provided valuable genetic resources that will facilitate the breeding of citrus varieties with improved waterlogging tolerance.

## Introduction

Citrus is one of the most economically and socially important evergreen fruit tree species globally. Waterlogging is a serious impediment to citrus production because it has significantly affected their yield, quality, as well as geographical distribution (García-Sánchez et al., 2007; Sampaio et al., 2021). Exposure to waterlogging may damage the root and could decrease the chlorophyll and cause leaf senescence (Pan et al., 2021; Ren et al., 2023; Tahjib-Ul-Arif et al., 2023). Hypoxia brought by the stress is becoming one of the crucial stress factors negatively affecting citrus production (Xie et al., 2021). Citrus is cultivated by grafting, with the rootstock being the organ that initially senses and responds to low oxygen conditions, thereby being seriously damaged by soil hypoxia (Ghaffari et al., 2021; Xie et al., 2021). Although changes in protein, gene expression, and metabolite levels have been studied under hypoxic stress, scarcely attention has been paid for the molecular responses to waterlogging stress in citrus.

Plants can temporarily maintain energy production during hypoxia caused by waterlogging (Gong et al., 2020; Pan et al., 2021). However, the anoxic metabolism in root forms toxic substances, including organic acids, excessive ethanol, and aldehydes, along with increased formation of reactive oxygen species (ROS) (Cheng et al., 2016). These inhibit the root function and plant growth, thus eventually causing cell death and leaf senescence (Teoh et al., 2022). During this complex biological process, numerous genes are activated, which are important for plant survival (Voesenek and Bailey Serres, 2015). For instance, transcription factors (TFs) are thought to control many waterlogging-responsive genes by directly binding to the appropriate stress-responsive cis-acting elements in the promoter regions (Christianson et al., 2010). The ERF transcription factors directly regulate waterlogging stress response, e.g., *PhERF2* expression was found to be upregulated in petunia (Yin et al., 2019), while *TaERFVII.1* expression alleviated the negative effects of waterlogging stress in wheat (Wei et al., 2019). Additionally, transcriptomic analysis of roots of kiwifruit (Zhang et al., 2015), citrus (Xie et al., 2021), and wheat (Shen et al., 2020) showed that numerous genes involved in photosynthesis, hormone production and signaling pathways, and ROS generation or scavenging etc. are expressed in response to waterlogging stress.

Plants can develop multiple adaptation strategies against waterlogging stress, including adventitious root formation, petiole elongation, and secondary aerenchyma development (Hattori et al., 2009; Bailey-Serres et al., 2012; Tahjib-Ul-Arif et al., 2023). Gene expression and signal transduction are also critical for plant survival under waterlogging stress (Liu et al., 2021). Currently, studies are being conducted on how citrus rootstocks respond to flooding stress, such as Xie et al. (2021) studied the transcript levels of rootstock and identified 232 hypoxia-responsive genes. However, the difference in transcript levels between the aboveground and belowground parts in citrus rootstock remain unclear. In this study, we performed a transcriptome profiling, and validated the morphological, physiological and biochemical to investigated the stress response of two waterlogging-tolerant citrus varieties, i.e., a new citrus rootstock called *Citrus junos* Sieb ex Tanaka cv. Pujiang Xiangcheng (Fu et al., 2017) and *C. junos* Ziyang Xiangcheng, along with one waterlogging-sensitive variety red tangerine to waterlogging stress. The objectives of this paper were to: (1) explore how waterlogging affects the plant growth of citrus rootstock cultivars with different waterlogging tolerances, and (2) investigate the underlying physiological and molecular mechanisms of the differences in waterlogging tolerance between the three citrus rootstock cultivars.

## Material and methods

### Plant materials and treatments

In this study, the cultivars named *C. junos* Sieb. Ex Tanaka cv. ‘Pujiang Xiangcheng’ (abbreviated Pj), *C. junos* ‘Ziyang Xiangcheng’ (abbreviated Zy) and red tangerine (*Citrus reticulata* Blanco, abbreviated Rt) were grown in an experimental orchard at Sichuan Citrus Germplasm Repository in Chengdu, China were used as materials. The mature fruits were harvested from the same tree, and the seeds were collected. Isolated seeds were surface sterilized using 0.5 M NaOH. The sterilized seedlings were then selected based on a uniform size and subsequently grown in wetted soil-containing pots in a growth chamber. All seedlings were cultured for approximately six months with normal watering and fertilizing regimen.

Three biological replicates (32 seedlings per replicate) were randomly selected for each treatment. Pots were then filled with water until the water level was ~1 cm above the soil level. Water was maintained at this level for the entire duration of the treatment. For control treatments, plants were watered every three days. On day 15 and 35 of the experiment, the seedlings with at least three fully expanded leaves were taken, and their SPAD value, shoot height, root length, and root numbers were measured.

### Determination of the physiological and biochemical indexes

The activities of antioxidant enzymes, including superoxide dismutase (SOD, EC 1.15.1.1), guaiacol peroxidase (POD, EC 1.11.1.7) and catalase (CAT, EC 1.11.1.6) were determined according to Shahid et al. (2019). Soluble sugar and protein were analyzed from leaves according to the previously described method (Zaher-Ara et al., 2016). The hydrogen peroxide (H_2_O_2_) and malondialdehyde (MDA) contents were determined by a spectrophotofluorometric method (Muñoz et al., 2008).

### RNA-seq, data processing, and expression analysis

Based on the research conducted by Xie et al. (2021), and daily field observations, all three rootstocks showed symptoms of waterlogging stress after processing treatment for 35 days. Then, the leaves and roots of Pj, Zy, and Rt were collected and stored at -80 °C. Small and equal amount of leaves from five plants were pooled together as one replicate. Three independent replicates were subjected to RNA extraction as described previously (He et al., 2018; He et al., 2022). Clean reads were submitted to the China National GeneBank DataBase (CNGBdb) database sequence read archive, under the project: CNP0004172. Before sequence assembly, the adapter sequences and low-quality reads were removed from the raw data (**Supplementary Table S1**). TopHat2 (Kim et al., 2013) was used to map the clean reads to the *C. junos* reference genome (unpublished). The number of fragments per kilobase of transcript per million mapped reads (FPKM) was calculated using the RSEM tool (Li and Dewey, 2011). The average FPKM values of the three replicates were taken as the expression level of genes in each sample. The sets of DEGs (differentially expressed genes) were identified using the eBays function in the limma package with |log2 (foldchange)| ≥ 1.0 and the adjusted *p*-value < 0.05.

A Weighted Gene Co-expression Network Analysis (WGCNA) was applied to evaluate the gene expression (Langfelder and Horvath, 2008). The flashClust toolkit (R language) was used to perform cluster analysis on samples and set appropriate thresholds (Müllner, 2013). Heatmaps were created with the heatmap2 function in the R environment.

### qRT-PCR analysis

To verify the authenticity of the transcriptional data, 13 key TFs identified in the leaf or root were selected for further qRT-PCR validation. Total RNA was extracted and purified using the EasyPure^®^ Plant RNA Kit (TransGen Biotech Co., Ltd., Beijing, China), as per the manufacturer’s protocol. The qRT-PCR mixture included 1 μL cDNA template, 5 μL of 2×*TransStart* ^®^ Green qPCR SuperMix (TransGen Biotech Co., Ltd., Beijing, China), and 0.5 μL each of reverse and forward primer. The PCR program was as described previously (He et al., 2018). The gene-specific primers used are shown in **Supplementary Table S2**. Three biological and three technical replicates were adopted. The 2^-△△Ct^ method was utilized to calculate the relative gene expression, with the *EF-1α* gene being used as the internal control (He et al., 2021).

### Statistical analysis

The collected data was prepared with Microsoft Excel. Significant differences between grafted combinations were analyzed by Tukey’s method, while Pearson correlation analysis and principal component analysis (PCA) were performed using SPSS 20.0 software (IBM, USA). Figures were drawn in the GraphPad Prism software (v. 7.04).

## Results

### Effects of waterlogging stress on phenotypic and physiological index

**Figure 1:**
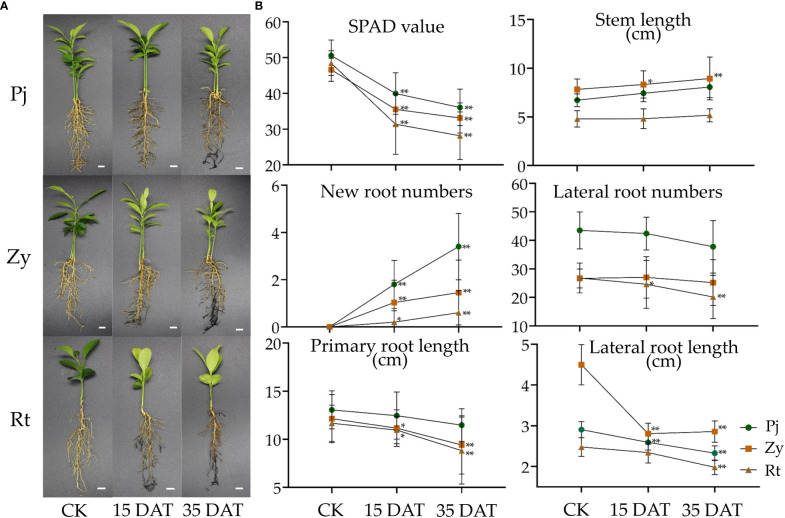
Phenotypic and physiological analysis of citrus rootstocks in response to waterlogging stress. **(A)** The phenotype of three citrus rootstocks under waterlogging stress. **(B)** The physiological analysis of citrus rootstocks in response to waterlogging stress. The bars represent the standard errors of the means (n ≥ 3). The asterisks indicate that the values are significantly different (* for *p* < 0.05 and ** for *p* < 0.01). Bars = 1 cm. Pj, Pujiang Xiangcheng (*Citrus junos* [Sieb.] Tanaka); Zy, Ziyang Xiangcheng (*C. junos* [Sieb.] Tanaka); Rt, Red tangerine (*Citrus reticulata* Blanco); DAT, Days after Treatment.

As shown in **Figure 1A**, waterlogging stress-induced root injury was observed in all the citrus rootstocks at 35 Days After Treatment (DAT). Additionally, the SPAD value of leaves, stem length, new root numbers, lateral root numbers, primary root length, and lateral root length were quantified post waterlogging stress or under control conditions (**Figure 1B**). The results indicated that Pj exhibited significant difference in its SPAD value, stem length, new root numbers, and lateral root length as compared with Zy and Rt (**Figure 1B**). Based on those data, we demonstrated that Pj was highly tolerant to waterlogging stress as compared with Zy and Rt.

### Effects of waterlogging stress on physiological and biochemical parameters

**Figure 2:**
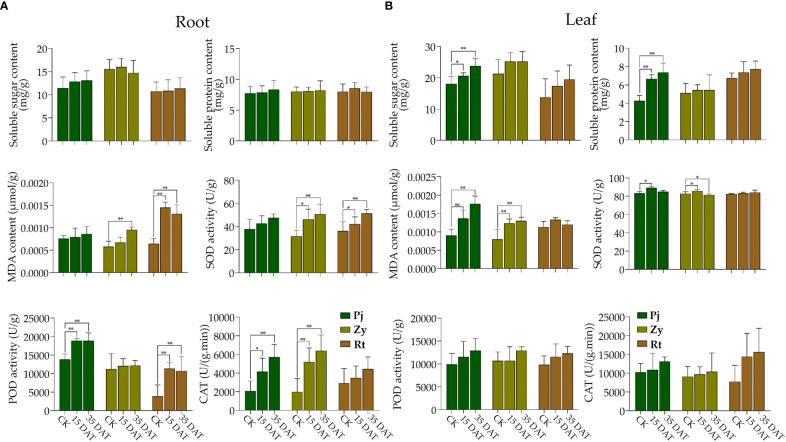
Effects on physiological and biochemical parameters of citrus rootstocks under waterlogging stress. **(A, B)** indicate the physiological and biochemical parameters of the root and leaf, respectively. The asterisks indicate that the values are significantly different (** for *p* < 0.01 and * for *p* < 0.05).

Waterlogging stress significantly affected the MDA content and SOD activity in the roots of Zy and Rt (**Figure 2A**). The treatment also induced noticeable changes in the POD activity in the roots of Pj and Rt, and the CAT activity in the roots of Pj and Zy, respectively (**Figure 2A**). However, the pattern of the effect of waterlogging stress on the soluble sugar and soluble protein contents in the roots of all rootstocks remained consistent with the control (**Figure 2A**).

Waterlogging stress significantly affected the accumulation of soluble sugar and soluble protein contents in the leaves of Pj (**Figure 2B**). Furthermore, the MDA contents of both Pj and Zy at 15 DAT and 35 DAT were higher than those in the control (**Figure 2B**). Although there were changes in physiological parameters in leaves of Rt, none were significant (**Figure 2B**).

### Analysis of differentially expressed genes

To further understand the underlying molecular mechanisms of waterlogging tolerance, we performed RNA sequencing of the root and leaf to identify the waterlogging stress-responsive DEGs (**Supplementary Table S1**). At 35 DAT, there were 624, 882, and 1058 upregulated DEGs and 984, 960, and 986 downregulated DEGs in the leaves of Pj, Zy and Rt, respectively. Additionally, there were 629, 1358, and 1506 upregulated DEGs, and 971, 800, and 894 downregulated DEGs in the roots of Pj, Zy and Rt, respectively (**Supplementary Table S3**). After performing KEGG (Kyoto Encyclopedia of Genes and Genomes) analysis on these DEGs, we found that the enriched pathways were mainly photosynthesis-related in the leaves and secondary metabolite biosynthesis-related in the roots (**Supplementary Figure S1**).

**Figure 3:**
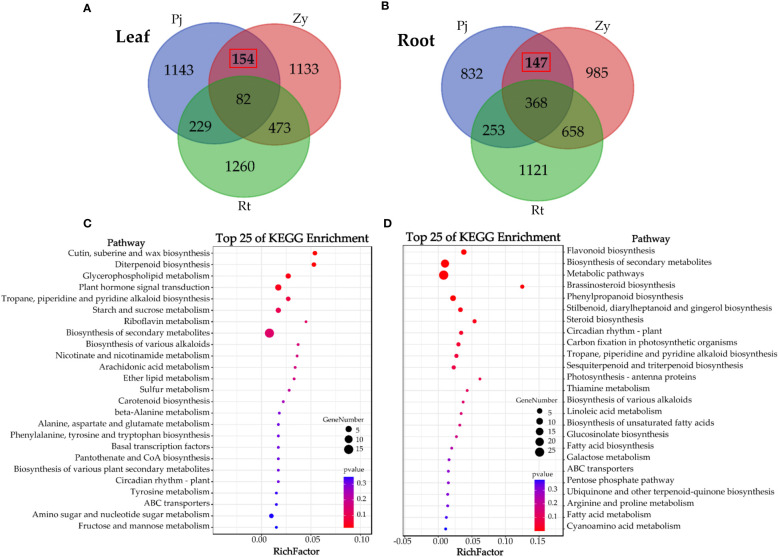
Analysis of the DEGs in the leaves and root of three rootstocks. **(A, C)** show the Venn diagram analyses of DEGs in leaves and KEGG enrichment analysis of 154 DEGs, respectively. **(B, D)** shows the Venn diagram analyses of DEGs in root and KEGG enrichment analysis of 147 DEGs, respectively.

To understand the difference in gene expression between *C. junos* and *C. reticulata* under waterlogging stress, we identified the DEGs and subjected them to KEGG enrichment analysis, and found 154 DEGs and 147 DEGs in the leaves and roots, respectively (**Figures 3A, B**). The KEGG analysis results showed that most DEGs in the leaves are those participated in the process of ‘cutin, suberine, and wax biosynthesis’, ‘diterpenoid biosynthesis’, ‘glycerophospholipid metabolism’ and ‘plant hormone signal transduction’ (*p* < 0.05, **Figure 3C**). Furthermore, the KEGG analysis results showed that most genes in the root participated in the ‘flavonoid biosynthesis’, ‘biosynthesis of secondary metabolites’, ‘metabolic pathways’, ‘brassinosteroid biosynthesis’, ‘phenylpropanoid biosynthesis’, ‘stilbenoid, diarylheptanoid and gingerol biosynthesis’, ‘steroid biosynthesis’, ‘circadian rhythm – plant’, and ‘carbon fixation in photosynthetic organisms’ (*p* < 0.05, **Figure 3D**).

### DEGs involved in ROS generation/scavenging and hormonal pathways

**Figure 4:**
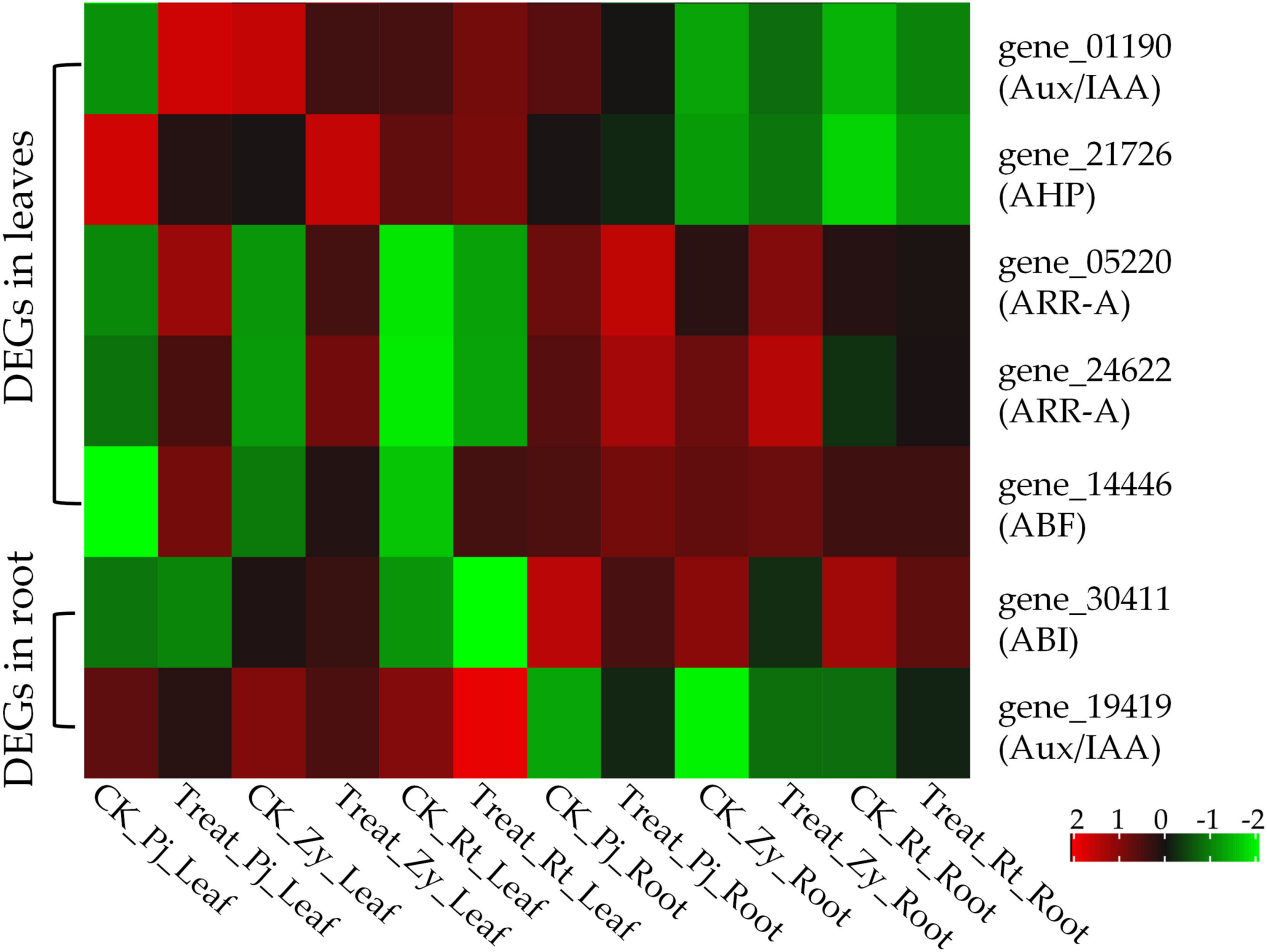
Heatmap of DEGs involved in ROS generation/scavenging and hormones. Heat map showing expression of the DEGs based on $\log_{10}^{\left(\text{FPKM}+0.01\right)}$ values, the same as below.

Typically, waterlogging causes ROS accumulation, which is mainly attributed to the coordinated expression of ROS generation/scavenging-related genes. In this study, epoxide hydrolase (gene_07329) genes were significantly upregulated by waterlogging stress in Pj and Zy, as compared with Rt (**Figure 4**). Among those DEGs in the leaves, five genes were enriched in ‘plant hormone signal transduction’ pathway, including one auxin/indole-3-acetic Acid (Aux/IAA, gene_01190), three cytokinine related genes (one histidine-containing phosphotransfer protein, gene_21726; two two-component response regulators, gene_05220 and gene_24622) and one ABA-responsive element binding factor (ABF, gene_14446) (**Figure 4**). However, there were no ROS-related DEGs in the root. But two hormone signal transduction related DEGs, including ABSCISIC ACID-INSENSITIVE 5-like protein (ABI, gene_30411) and Aux/IAA (gene_19419), were identified in the roots (**Figure 4**). Therefore, ABF was upregulated, while Aux/IAA was downregulated in all the rootstocks post waterlogging treatment for 35 days.

### Differentially expressed transcription factors

**Figure 5:**
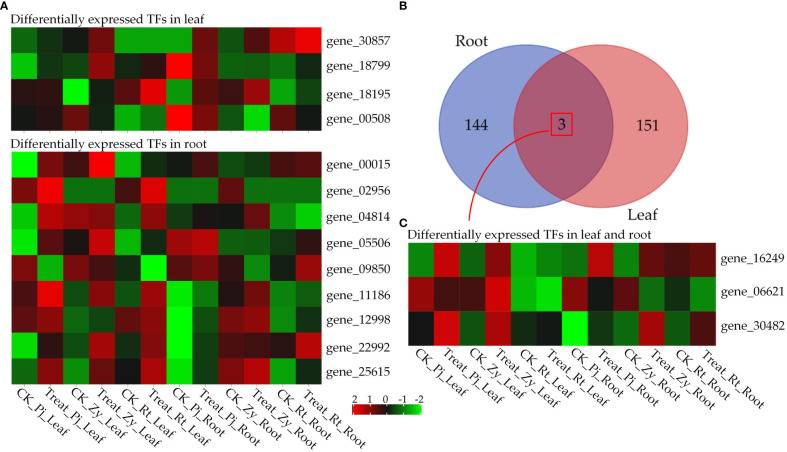
Differentially expressed transcription factors. **(A)** Heatmap of tissue-specific transcription factors. **(B)** Venn diagram analyses of DEGs in leaf and root. **(C)** Heatmap of differentially expressed transcription factors in both leaf and root.

In the shoot, six TFs, including an ethylene response factor (AP2/ERF, gene_00508), a *bHLH* (basic helix-loop-helix, gene_18799), a *HSF* (Heat Stress Transcription Factor, gene_30482) and three unkown TFs (gene_18195, gene_16249 and gene_30857) were differentially expressed between *C. junos* and *C. reticulata* (**Supplementary Table S4**). In the root, 11 differentially expressed TFs between genotypes were identified, including two HSFs, one *EREBP* (ethylene-responsive element binding protein), three WRKY transcription factor and five unkonw TFs (gene_04814, gene_25615, gene_11186, gene_16249 and gene_09850) (**Supplementary Table S5**). Overall, 16 of 17 TFs were upregulated in treated sample compared with controls (**Figure 5A**). It is worthy to note that *EREBP* (gene_02956) only expressed in the roots of Pj and Zy, and gene_30857 only expressed in the leaves of Zy after treatment (**Figure 5A**). All the differentially expressed TFs were tissue-specific, except gene_16249 and gene_30482 (**Figures 5B, C**). In details, gene_16249 and gene_30482 were upregulated in three rootstocks (**Figure 5C**).

### Weighted gene co-expression network analysis

**Figure 6:**
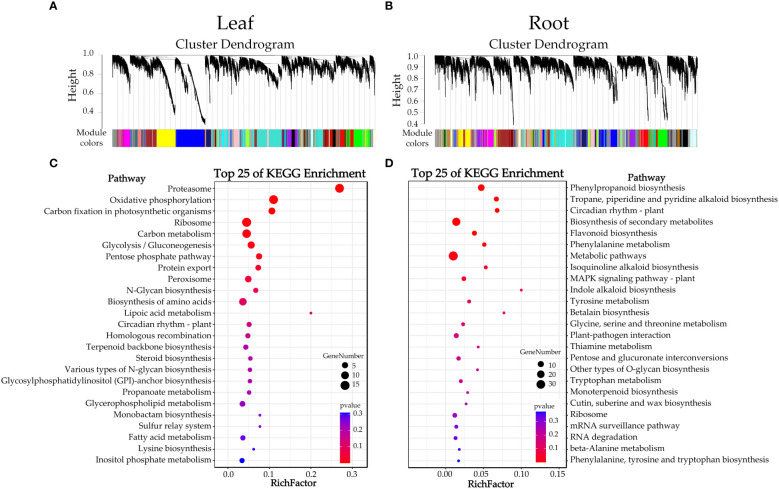
Clusters dendrograms and KEGG enrichment analysis. **(A, B)** indicate the merged clusters and dendrograms, **(C, D)** indicate the KEGG enrichment analysis of genes in the ‘salmon’ and ‘skyblue’ modules, respectively.

To identify the co-expression modules correlated to waterlogging tolerance and the hub genes involved in their transcriptional regulatory networks, we carried out a weighted gene co-expression network analysis (WGCNA). Our results revealed 30 and 37 co-expressed modules in the leaf and the root, after gathering from 28 to 4168 and from 44 to 2132 genes, respectively (**Figures 6A, B** and **Supplementary Figure S2**). Additionally, we also analyzed the correlation between these modules with seven metabolites (soluble sugar content, soluble protein content, MDA content, SOD activity, POD activity, CAT and H_2_O_2_ content). The results showed that the ‘salmon’ module was significantly positively correlated with the H_2_O_2_ content in the leaf (*r* = 0.83), whereas the ‘skyblue’ module was significantly positively correlated with the POD activity in the root (*r* = 0.79). Therefore, this result suggested that the 441 and 206 genes in the ‘salmon’ and ‘skyblue’ modules, respectively, may play important roles in the waterlogging tolerance of citrus (**Supplementary Tables S6, S7**). Furthermore, we utilized the KME (eigengene connectivity) value to determine the hub genes in the ‘salmon’ module in the leaf and the ‘skyblue’ module in the root. This analysis allowed us to identify gene_23569 (NADH dehydrogenase (ubiquinone) 1 alpha subcomplex subunit 5) as a hub gene within the ‘salmon’ module, and gene_13979 (function is currently unknown) as a hub gene within the ‘skyblue’ module. These hub genes may play important roles in regulating the expression patterns of other genes within their respective modules.

We also performed KEGG enrichment analysis for genes in the ‘salmon’ and ‘skyblue’ modules. The results showed that genes in the ‘salmon’ module participated in the ‘proteasome’, ‘oxidative phosphorylation’ and ‘carbon fixation in photosynthetic organisms’ pathways (top three pathways of KEGG enrichment) (**Figure 6C**). Furthermore, the genes in the ‘skyblue’ module participated in the ‘phenylpropanoid biosynthesis’, ‘tropane, piperidine, and pyridine alkaloid biosynthesis’ and ‘circadian rhythm – plant’ pathways (top three pathways of KEGG enrichment) (**Figure 6D**).

### Validation of candidate genes by qRT-PCR analysis

**Figure 7:**
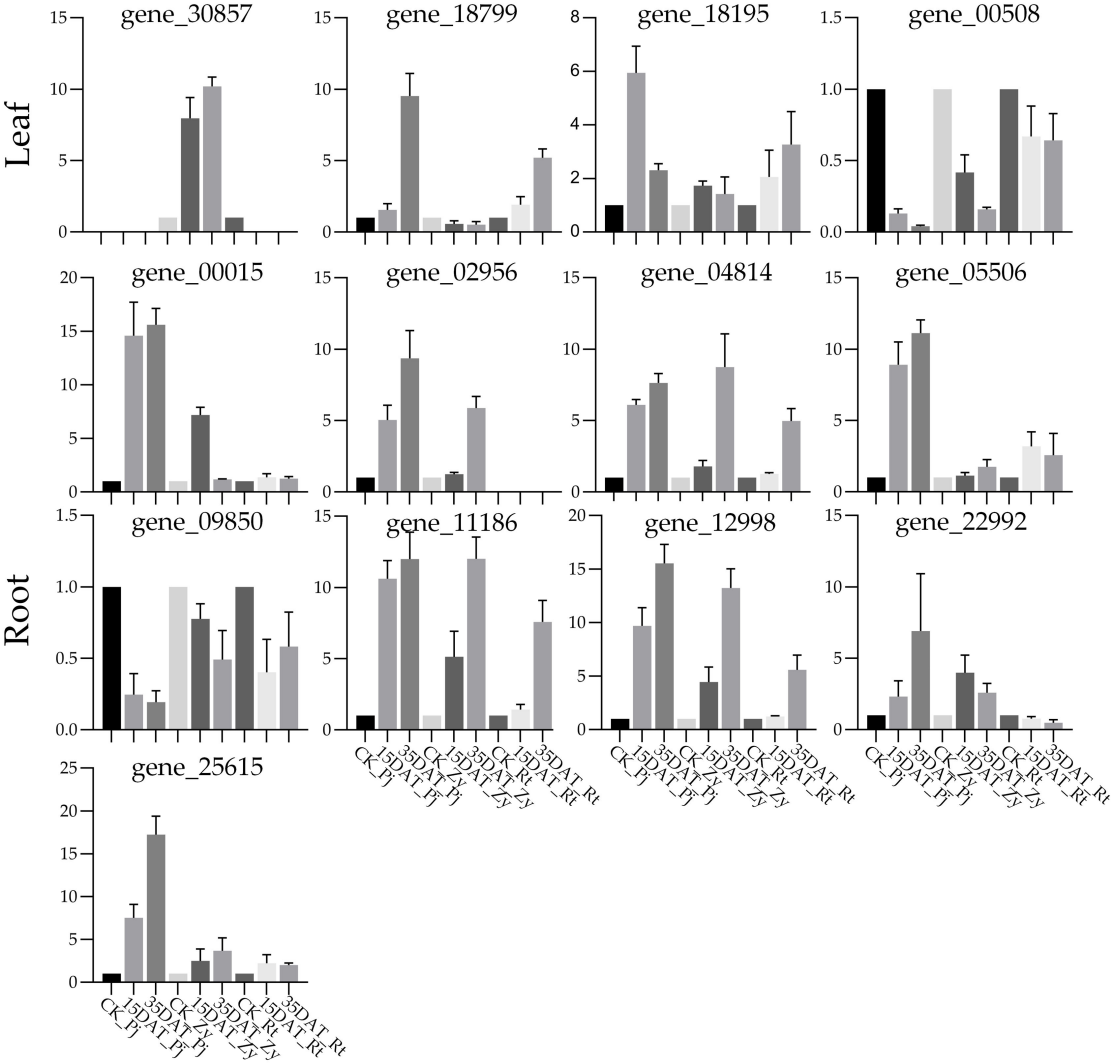
Verification of the expression of eight candidate genes’ in different rootstocks. The error bars with standard deviations are calculated from three biological replicates.

To validate the accuracy of the RNA-Seq expression patterns, we have chosen 13 key candidate TFs for qRT-PCR validation. The results showed that the qRT-PCR expression levels were generally consistent with RNA-Seq data, with a good positive correlation (*R* ^2 =^ 91.92) (**Supplementary Figure S3**), thereby confirming the transcriptome data reliability in the present study. Among those genes, *EREBP* (gene_02956) was only expressed in the roots of Pj and Zy, while gene_30857 only expressed in the leaves of Zy after treatment (**Figure 7**).

## Discussion

### Physiological and morphological response to waterlogging stress

Field observations of waterlogging responses in citrus have shown that the roots of Rt suffered greater damage than those of Pj and Zy (**Figure 1A**). In this study, the leaf chlorophyll content detected by SPAD in Pj showed the smallest reduction as compared to those of the other two rootstocks. Additionally, waterlogging stress slightly increased the stem length (**Figure 1B**), which is consistent with previous results in soybean (Kim et al., 2015). Waterlogging stress also affected plant growth and development, thereby causing morphological developments, including aerenchyma formation, adventitious root formation, and shoot elongation (Qi et al., 2019). Here, all rootstock seedlings under waterlogging stress showed increased adventitious root formation, with Pj being significantly higher than in other rootstocks (**Figure 1B**). Stress triggers diverse plant responses, including physiological and metabolic process (Zhang, H. et al., 2020; Zhang, H.M. et al., 2022). A series of antioxidant enzymes generated following ROS accumulation in plant, helped eliminate the excess ROS. In this study, the soluble sugar, soluble protein, and MDA contents increased in the leaf, while the POD and CAT activities increased in roots of stress tolerant citrus, respectively (**Figure 2**). Therefore, these might keep ROS at a low level in Pj.

### Gene transcription changes in citrus under waterlogging stress

The waterlogging stress-responsive molecular and physiological events have been reported in diverse plant species (Pan et al., 2021). Recent studies focusing on citrus rootstocks have revealed that hypoxia stress can lead to a significant decrease in mineral element contents and identification of 232 hypoxia-responsive genes (Xie et al., 2021). However, there is still limited knowledge about the underlying waterlogging stress-responsive molecular mechanisms in citrus under waterlogging stress. In this study, there were 154 DEGs in the leaves and 147 DEGs in the root after comparing the gene expression of three rootstocks. Typically, a range of energy-related and oxygen-consuming metabolic pathways were differentially regulated to mitigate the harmful effects of waterlogging stress (Blokhina and Fagerstedt, 2010). In this study, numerous genes related to ‘carbon fixation in photosynthetic organisms’ in the root (**Figure 3D**).

Hormone has long been known to be involved in waterlogging stress responses. Several genes associated with auxin and the ABA pathway were found to have a significantly expressed in response to waterlogging stress. ABA has been regarded as being closely related to water stress (Zhang, Q. et al., 2022). We identified one ABSCISIC ACID-INSENSITIVE 5-like protein (ABI, gene_30411) in the root, which confers hypersensitivity to ABA and sugar (Brocard et al., 2002). Additionally, one ABA-responsive element binding factor (ABF, gene_14446) was differentially expression in leaf (**Figure 4A**). ABA is able to integrate auxin signaling to modulate plant performance under different stress conditions (Emenecker and Strader, 2020). There were two different expression Auxin/Indole-3-Acetic Acid genes in the leaf (gene_01190) and root (gene_19419), respectively. Auxin/IAA is induced by auxin and together with *SAUR* and *GH* family genes, and they are collectively referred to as early auxin-induced genes (Yu et al., 2022). Moreover, we identified three cytokinin-related genes (one histidine-containing phosphotransfer protein, gene_21726; two two-component response regulators, gene_05220 and gene_24622) in the leaf, which have been previously associated with plant growth and stress tolerance (Merewitz et al., 2010).

### Transcription factors involved in waterlogging stress

Several types of TFs have been shown to be involved in abiotic stresses. Two specific TFs, namely *MxWRKY64* and *MxbHLH18*, which are extracted from *Malus xiaojinensis*, have been found to play an effective role in enhancing salt tolerance (Han et al., 2021; Liang et al., 2022). Moreover, four other TFs, *MbMYB4* and *MbMYB108* derived from *Malus baccata*, and *ERF9* and *ERF108* derived from *Poncirus trifoliata* have been proven useful in elevating cold tolerance (Khan et al., 2021; Yao et al., 2022a; Yao et al., 2022b; Zhang, Y. et al., 2022). Through various research results, it has been increasingly apparent that such TFs can play a significant role in improving plant resilience against waterlogging stress. *MaRAP2-4* showed enhanced waterlogging and subsequent oxidative stress tolerance (Phukan et al., 2018). RNA-seq results also revealed the involvement of WRKY, MYB, bHLH, NAC, ERFs, DOF, HD-ZIP and DBP in hypoxic stress (Xie et al., 2021; Wang et al., 2023). In this study, 16 TFs, including AP2/ERFs, bHLHs, HSFs and WRKYs, were found to be associated with waterlogging stress. However, due to the limited data available on these waterlogging-responsive TFs, their functions need to be intensively dissected in the future.

### Underlying mechanisms of waterlogging tolerance in citrus

**Figure 8:**
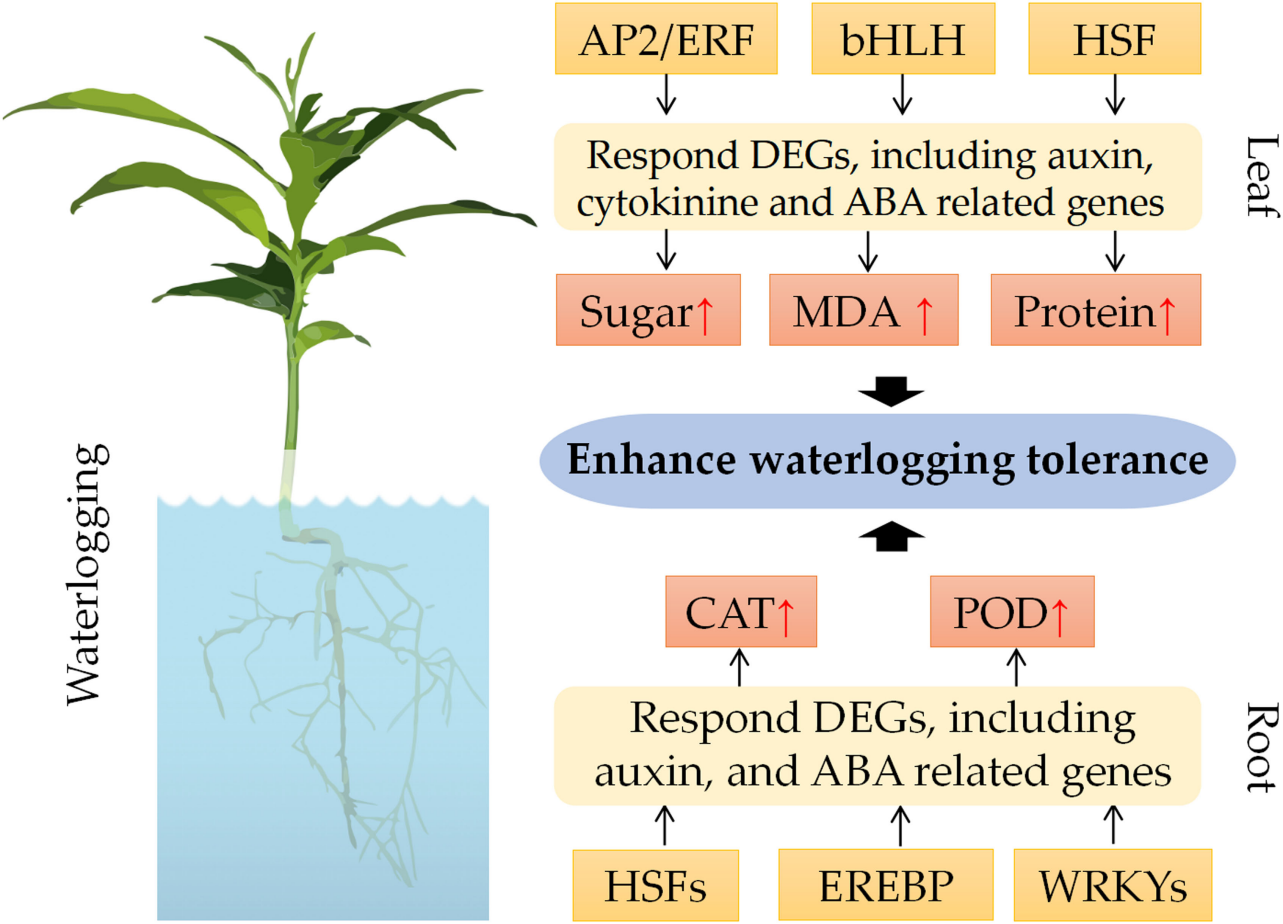
Proposed model for the waterlogging tolerance in *Citrus junos*.

To elucidate the molecular basis of waterlogging tolerance in *C. junos*, we developed a working model based on our results (**Figure 8**). According to this model, the transcription factors AP2/ERF, bHLH and HSF may modulate the expression of DEGs involved in auxin, cytokinine and ABA pathways in leaves (Zhao et al., 2020; Li et al., 2022; Li et al., 2023). This may lead to physiological and biochemical adjustments, such as enhanced sugar content, protein content and MDA content. Similarly, the transcription factors HSFs, EREBP and WRKYs may regulate the expression of DEGs in roots (Zhang, C. et al., 2020; Liu et al., 2021). These differentially expressed TFs may alter metabolic processes, such as elevating CAT activity and POD activity. These changes may ultimately confer waterlogging tolerance to citrus plants.

## Conclusions

Waterlogging stress negatively affected citrus growth, which decreased the SPAD value and root number. Under waterlogging stress, the soluble sugar, soluble protein, and MDA contents in the leaf increased, while the CAT and POD activities increased in the root. RNA-seq analysis identified 154 DEGs in the leaves and 147 DEGs in the root. Processes like ‘cutin, suberine and wax biosynthesis’, ‘diterpenoid biosynthesis’, and ‘glycerophospholipid metabolism’ were enriched in the leaf, while those linked to ‘flavonoid biosynthesis’, ‘biosynthesis of secondary metabolites’, and ‘metabolic pathways’ were seen in the root. Furthermore, we identified 17 differentially expressed TFs, which mainly are important in the waterlogging tolerance of citrus. Therefore, we proposed the working model of waterlogging tolerance based on those evidences. Our findings will deepen our understandings for the mechanism of waterlogging tolerance in citrus.

## Data availability statement

The datasets presented in this study can be found in online repositories. The names of the repository/repositories and accession number(s) can be found in the article/**Supplementary Material**.

## Author contributions

Conceptualization and supervision, WH and XW. Methodology, WH and LL. Investigation, LL, ZW, SY, ML, YL, YZ, YXL, YTZ, and HW. Bioinformatic analyses, WH and QC. Data curation, WH, LL, RX, and JC. Manuscript preparation, WH and YW. Writing-review and editing, WH, LL, and XW. All authors contributed to the article and approved the submitted version.
