# Supplementary material for: Transcriptome sequencing analyses uncover mechanisms of citrus rootstock seedlings under waterlogging stress
Source: Front Plant Sci. 2023 May 31;14:1198930. doi: 10.3389/fpls.2023.1198930 (PMC10264899; doi:10.3389/fpls.2023.1198930)
Supplement: Supplementary file 1 [file DataSheet_1.pdf]

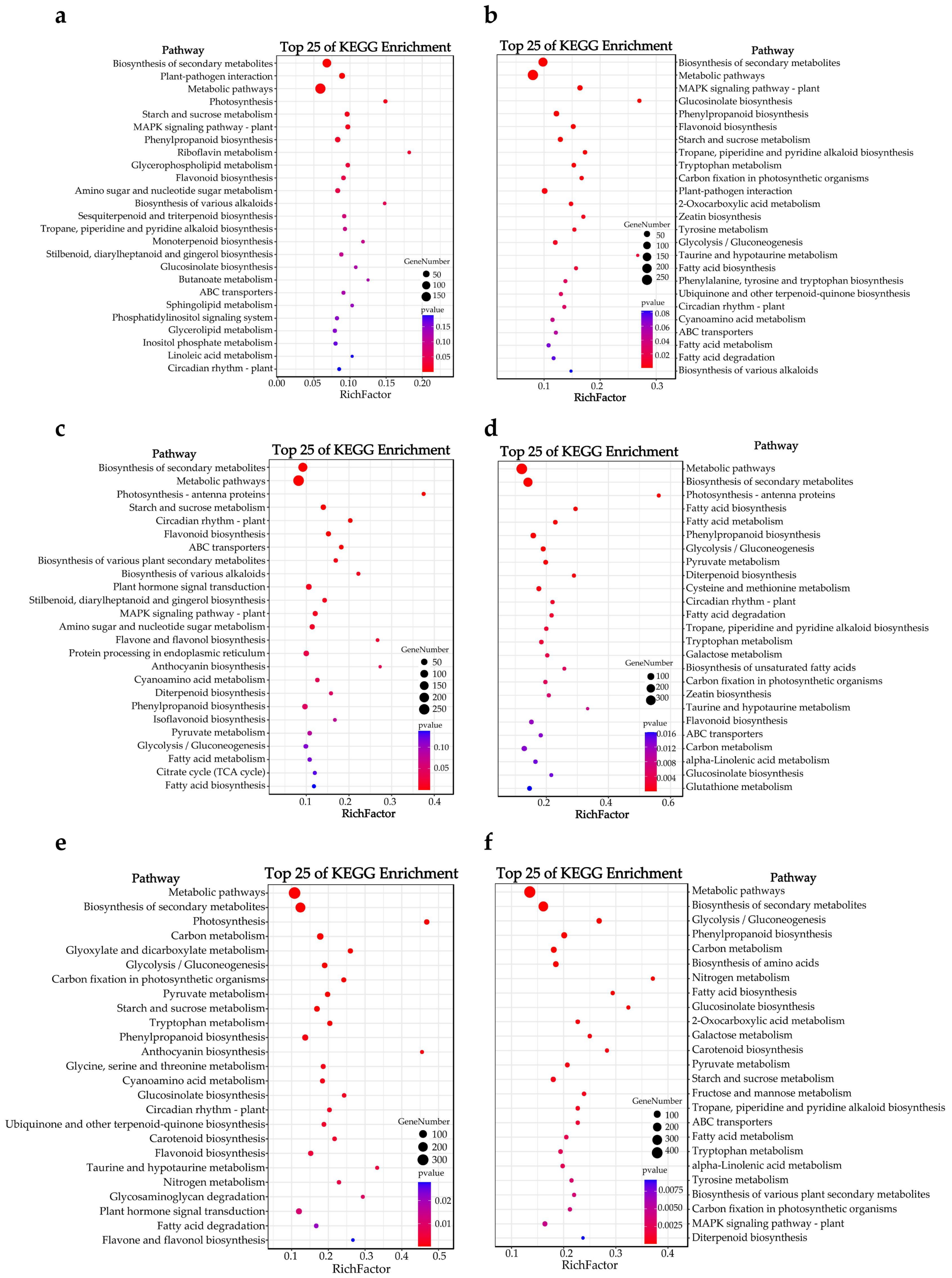

Figure S1. KEGG metabolism pathway categories of DEGs. (a) Pj leaf, **(b)** Pj root, **(c)** Zy leaf, **(d)** Zy root, **(e)** Rt leaf, **(f)** Rt root

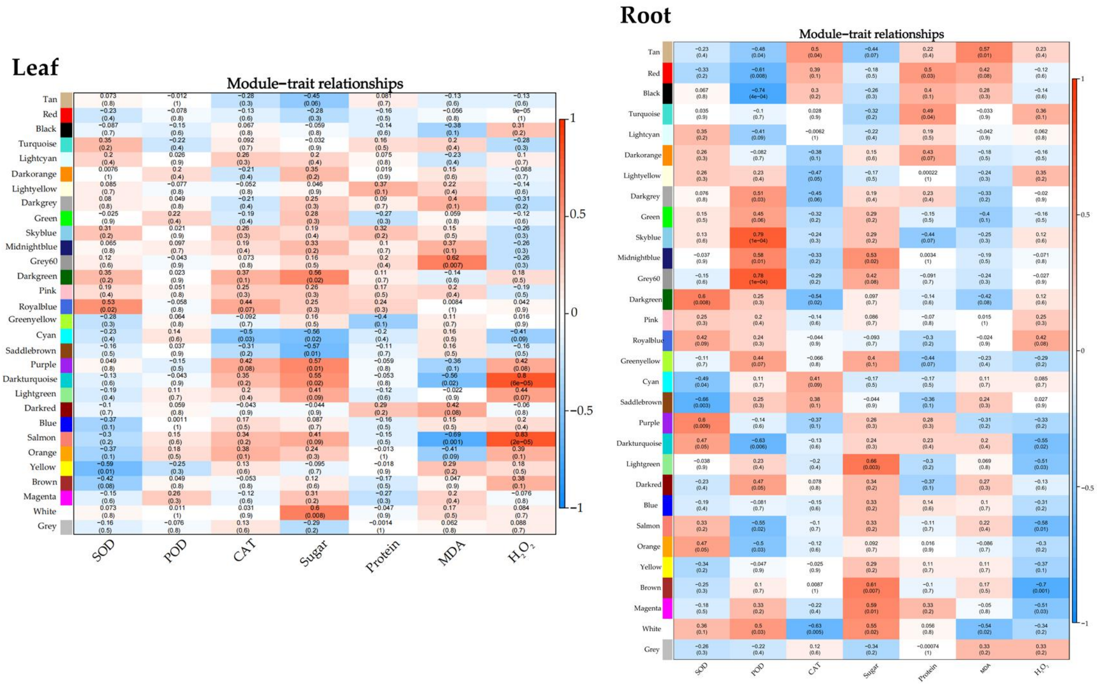

Figure S2. Module-trait relationships were obtained through the WGCNA analysis in leaf and root.

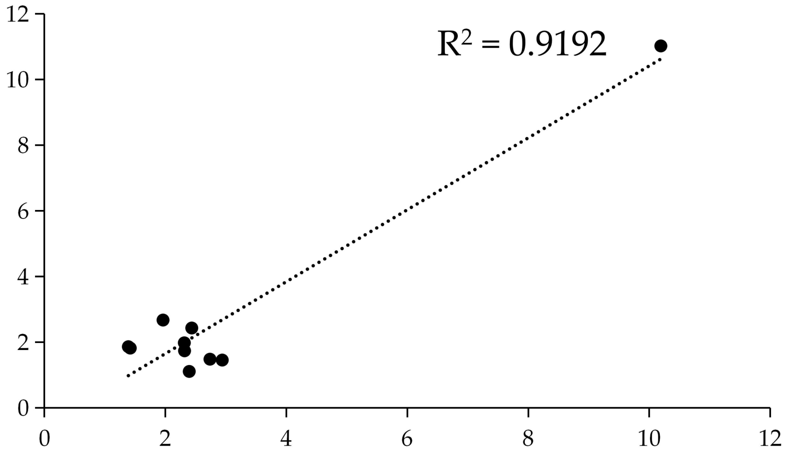

Figure S3. Pearson's correlation of RNA-seq and qRT-PCR results.
